# Study on Rumen Degradability and Intestinal Digestibility of Mutton Sheep Diets with Different Concentrate-to-Forage Ratios and Nonfiber Carbohydrates/Neutral Detergent Fiber Ratios

**DOI:** 10.3390/ani14192816

**Published:** 2024-09-29

**Authors:** Xunyu Guo, Lei Sun, Zibin Zheng, Xiaogao Diao, Liwen He, Xiaoling Dong, Wei Zhang

**Affiliations:** 1 State Key Laboratory of Animal Nutrition and Feeding, College of Animal Science and Technology, China Agricultural University, Beijing 100193, China; cauguoxunyu@163.com (X.G.); zhengzibin@cau.edu.cn (Z.Z.); helw@cau.edu.cn (L.H.); 2 Beijing DaBeiNong Technology Group Co., Ltd., Beijing 100095, China; sl641432395@126.com; 3 Sanya Institute, China Agricultural University, Sanya 572025, China; b20193040335@cau.edu.cn

**Keywords:** dietary concentrate-to-forage ratio, NFC/NDF, rumen degradability, intestine digestibility, mutton sheep

## Abstract

**Simple Summary:**

At present, indoor feeding is the primary method for fattening mutton sheep in China, where high-concentrate feeding is the common way to achieve high growth performance in the short term, and dietary cost accounts for most of the feeding expenditure. However, increasing feed costs and metabolic diseases, like rumen acidosis and urinary calculi, are likely to compromise the production benefits of such a fattening mode. Exploring low-cost alternative feeds and optimized dietary concentrate levels would contribute to better production benefits. In this study, an in situ method and in vitro three-step method were used to investigate the optimum dietary concentrate-to-forage ratio, NFC/NDF (Nonfiber Carbohydrates/Neutral Detergent Fiber) ratio, and raw materials combination, ultimately providing a reference for the efficient and high-quality fattening of mutton sheep. It is concluded that a dietary concentrate-to-forage ratio in the range of 70:30~80:20 and NFC/NDF of 1.5~2.0 are recommended for fattening mutton sheep, and it is feasible to partly substitute soybean meal with unconventional protein feedstuff like cottonseed meal and rapeseed meal. In addition, the nutritional values of sunflower seed hulls and rice hulls are lower than that of peanut vine for mutton sheep. Such a study is of great significance to the high-quality development of the mutton sheep industry.

**Abstract:**

This study was conducted to investigate the rumen degradability and intestinal digestibility of mutton sheep diets different in concentrate-to-forage ratio, NFC/NDF, and ingredient combination, providing a guideline for the selection of a fattening diet for mutton sheep. Twenty-eight diets composed of four raw material combinations and seven concentrate-to-forage ratios and four three-year-old mutton sheep with permanent rumen fistulas were used in the experiments. The nutrient composition of the diets was first analyzed, and then an in situ method and in vitro three-step method were separately used to measure the rumen degradability and intestinal digestibility, mainly focusing on the effects of dietary concentrate-to-forage ratio and NFC/NDF as well as the effects of soybean meal and soybean meal replacement and peanut vine and peanut vine replacement. The results showed that a dietary concentrate-to-forage ratio of 70:30~80:20 and an NFC/NDF ratio of 1.5~2.0 are recommended for fattening mutton sheep, and low-cost cottonseed meal and rapeseed meal can be feasible alternative protein sources to soybean meal. In addition, the nutritional values of sunflower seed hulls and rice hulls for mutton sheep are lower than that of peanut vine. Such a study can provide practical guidelines for enterprises and farmers, being of important significance for the high-quality development of the mutton sheep industry.

## 1. Introduction

Mutton is highly palatable and possesses high nutritional value, making it an important source of protein for human nutrition [1]. The feeding of mutton sheep differs significantly from that of monogastric animals due to rumen physiology. In recent years, the mutton sheep industry has demonstrated a notable growth trend, accompanied by an intensifying interest among farmers in enhancing the productive performance of their herds. Traditionally, they were raised on grazing alone or grazing supplemented with a small amount of concentrate. However, in modern farming practices, the use of concentrated feed has increased significantly, particularly during the latter stages of the fattening process [2,3]. However, excessive concentrate can easily lead to diseases such as rumen acidosis and urinary calculi, affecting animal health, feed utilization, and animal product quality, and even causing economic losses [4]. Therefore, the appropriate concentrate level is crucial for the health, feed utilization, and economic efficiency of meat sheep [5]. The dietary concentrate-to-forage ratio and NFC/NDF (Nonfiber Carbohydrates/Neutral Detergent Fiber ratio) are commonly used to reflect the level of dietary concentrate, among which “concentrate-to-forage ratio“ is more commonly used in actual production, which roughly reflects the main source of dietary raw materials. The “NFC/NDF” ratio distinguishes the nutritional composition characteristics of the diet from the material structure, and the NFC/NDF can accurately represent the levels of different types of carbohydrates in the diet. High-NFC/NDF-ratio diets will reduce the rumen microbial and bacterial diversity of goats and mutton sheep, thereby affecting their rumen health [6,7]. Adjusting the precision ratio is a common method to change the level of NFC/NDF [8,9]. Understanding the changes in nutrient composition, rumen degradability, and intestinal digestibility characteristics of mutton sheep diets with different concentrate levels will help in selecting appropriate concentrate levels in mutton sheep production to obtain the best performance or maximum yield. Many researchers have studied the effects of different dietary concentrate levels on mutton sheep, but the concentration gradient set is small [10,11]. The in situ method is a commonly used method to evaluate the nutritional value and rumen degradation characteristics of feed. The in vitro three-step method simulates the temperature and pH of the small intestine. It involves enzymatic incubation of the feed in vitro and the measurement of the nutrient content in the incubation residues to calculate the small intestinal digestibility. Therefore, four groups of dietary and seven kinds of concentrate-to-forage ratios in all twenty-eight kinds of NFC/NDF ratios were set in this experiment. We used an in situ method and an in vitro three-step method to explore the rumen degradability and intestinal digestibility of multiple concentrate levels, in order to provide references for the selection of a dietary concentrate-to-forage ratio and NFC/NDF during the fattening period of mutton sheep.

In the process of production and breeding, the cost of the feed accounts for more than 50% of the production cost of mutton sheep [12]. The selection of high-quality, low-cost feed, and thus saving on feed costs, is crucial to the development of the mutton sheep industry. Research shows that compared to soybean meal, cottonseed and rapeseed meal are 40–60% less expensive and easier to obtain [13]. Cotton and oilseed rape agriculture is an excellent producer of quality protein and can, thus, function as a protein supplement in animal feed [14]. In addition, roughage is a crucial component of ruminant feeds; peanut vine is a by-product of peanuts, and it can be used as a quality roughage resource, but the price is high [15]. Rice is widely cultivated all over the world and is a principal food for more than half of the world’s people. Rice hulls are a by-product produced from the rice milling process, and a large amount of rice hulls are produced every year [16]. Rice hulls are also increasingly being added to the diets of ruminants by farmers [17,18]. Sunflower is one of the world’s leading oil crops, and is produced in great quantities in the Inner Mongolia region of China [19]. A lot of sunflower seed hulls are wasted during processing, and many local farmers already use sunflower seed hulls as forage in the diet of mutton sheep [20]. In this study, low-priced cottonseed meal and rapeseed meal were selected to replace soybean meal, and low-priced rice hulls and sunflower seed hulls were selected to replace peanut vine. Four dietary groups were created to study how well they degrade in the rumen and are digested in the intestine. This research will help in choosing the best feed materials for mutton sheep during their fattening period.

In this paper, what is being tested is the nutritional efficacy of different concentrate sources and forage sources in an increasing combination of concentrate-to-forage ratio in order to verify the variation, in the first case, and the reduction, in the second case, of the ruminal and intestinal digestibility of the ration in adult sheep.

## 2. Materials and Methods

All animal management and experimental procedures followed the animal care protocols approved by the Animal Care and Use Ethics Committee of China Agricultural University.

### 2.1. Animals and Diets

Four ruminally fistulated ZhaoWuDa castrated male sheep, aged 3 years old with similar weight (53.88 ± 1.65 kg) and in good health, were selected. The animals were fed in a single field twice daily at 7:00 and 17:00, with free access to clean water. The dietary composition and nutrient levels are as follows (Table 1).

### 2.2. Sample Preparation and Nutrient Analysis

The dietary raw materials used in this experiment were collected from Chifeng DaBeiNong Technology Group Co., Ltd. (Chifeng, China). The names of the four groups of diets and raw materials were designed as follows: SM (PV) group: soybean meal group (peanut vine group), raw materials: corn grain, soybean meal, DDGS, wheat bran, peanut vine, sodium bicarbonate, premix. NSM group: no soybean meal group, raw materials: corn grain, cottonseed meal, rapeseed meal, DDGS, wheat bran, peanut vine, sodium bicarbonate, premix. SSHP group: sunflower seed hull and peanut vine group, raw materials: corn grain, soybean meal, DDGS, wheat bran, sunflower seed hulls, peanut vine, sodium bicarbonate, premix. SSHR group: sunflower seed hulls and rice hulls, raw materials: corn grain, soybean meal, DDGS, wheat bran, sunflower seed hulls, rice hulls, sodium bicarbonate, premix. In addition, the SM (PV) group and NSM group were formulated according to the principles of soybean meal and soybean meal substitution. The SM (PV) group, SSHP group, and SSHR group were formulated according to peanut vine and peanut vine substitution.

According to the feeding standard of nutrient requirements of mutton sheep and goats (NY/T 816-2021), seven experimental diets were prepared according to the concentrate-to-forage ratios of 90:10, 85:15, 80:20, 75:25, 70:30, 65:35, and 60:40, comprising a total of twenty-eight experimental diets, the nutritional routine was determined, and the NFC/NDF was calculated (Table 2, Table 3, Table 4 and Table 5).

### 2.3. Experimental Design

The in situ degradability values of DM, CP, NDF, and ADF in the 28 diets were determined according to the procedure described by Mehrez and Ørskov [21]. We conducted this experiment using a split-plot design, performing the experiment every other day. Three grams of the experimental diet was taken and placed in a nylon bag of known weight. We prepared two bags for each mutton sheep, with four mutton sheep as four replicates. We closed each nylon bag securely with a rubber band, and then used a 3 mm diameter, 25 cm long semi-soft plastic tube to tie two bags together with nylon string. The experiment followed a “ simultaneous insertion and removal “ method. We inserted the bags into the rumen sac at 17:00 before feeding, and removed them 16 h later through the rumen fistula. We immediately rinsed the nylon bags with clean water.

We dried the cleaned nylon bags in an oven at 65 °C for 48 h to a constant weight. We cooled the bags to room temperature in a desiccator, then weighed the nylon bags and the residues. We stored the residues in sealed plastic bags. We used part of the rumen residues to determine the rumen degradability of DM, CP, NDF, and ADF, and the rest to determine the intestinal digestibility.

The intestinal digestibility values of DM and CP in the 28 diets were determined according to the procedure described by Gargallo [22]. One gram of rumen undegraded residue was taken and put into a nylon bag of known weight. We prepared two replicates for each mutton sheep. We placed the nylon bags in culture bottles containing a pepsin–HCl (hydrochloric acid) solution and incubated them in an ANKOM Daisy II in vitro fermentation incubator at 39 °C for 1 h. After 1 h, we removed and rinsed the nylon bags. We transferred the rinsed nylon bags to a trypsin solution and incubated them at 39 °C for 24 h. After 24 h, we removed the nylon bags, rinsed them, and then dried them in an oven at 55 °C for 48 h until they reached a constant weight. We cooled the bags to room temperature in a desiccator, weighed the nylon bags and residues, and stored the residues in sealed plastic bags. We determined the DM and CP contents in the residues to calculate the intestinal digestibility.

### 2.4. Solution Preparation

#### 2.4.1. Preparation of Hydrochloric Acid–Pepsin Solution

We took 9 mL hydrochloric acid (analytically pure, 12 mol/L) and added distilled water to 1000 mL, and prepared 0.1 mol/L HCl (pH = 1.9) solution. We added 1 g pepsin (P-7000, Sigma, Kawasaki, Japan) to this solution and mixed well [22].

#### 2.4.2. Preparation of Trypsin Solution

Liquid A: we measured out 179.07 g of disodium hydrogen phosphate (Na_2_HPO_4_·12H_2_O, analytically pure), dissolved it in distilled water, and set the volume to 1000 mL. Liquid B: we measured out 78.01 g sodium dihydrogen phosphate (NaH_2_PO_4_·2H_2_O, analytically pure), dissolved it in distilled water, and set the volume to 1000 mL. We mixed 915 mL of liquid A with 85 mL of liquid B, adjusted the pH to 7.75, and added 50 mg/L of thymol and 3 g/L of trypsin (P-7545, Sigma, Kawasaki, Japan) to the buffer [22].

### 2.5. Determination Index and Method

#### 2.5.1. Determination of Nutritional Routine

To prepare feed samples, raw materials were dried at 65 °C for 48 h in a forced-air oven and then milled through a 1 mm sieve for chemical analysis and a 2.5 mm sieve for in situ degradation. The contents of DM, CP, Ash, Ca, P, and EE were analyzed according to the methods of AOAC [23]. The contents of NDF and ADF were analyzed using an automatic fiber analyzer (A2000i, Ankom Technology, Macedon, NY, USA) following the methods described by Van Soest et al. [24].

#### 2.5.2. Calculation of Rumen Degradability and Intestinal Digestibility of Nutrients in the Measured Diet

Rumen Degradability and Intestinal Digestibility of Nutrients in the Measured Diet are calculated as:

$$rumen\;degradability\left(\%\right)=\frac{A-B}{A}\times 100$$

where A is the content of a nutrient before rumen degradation, and B is the content of a nutrient after rumen degradation.

$$intestinal\;digestibility\left(\%\right)=\frac{A-B}{A}\times 100$$

where A is the content of a nutrient before intestinal digestion, and B is the content of a nutrient after intestinal digestion.

### 2.6. Statistical Analysis

The data were calculated and processed by Excel 2019 and the table was drawn. SPSS 25.0 software (SPSS Inc., Chicago, IL, USA) was used for one-way ANOVA analysis, and the HSD method was used for multiple comparison testing; *p* < 0.05 was considered to indicate significance. All data are expressed in the form of mean ± SD. There was no interaction-among-ratios effect, concentrate effect, or forage effect (*p* > 0.05). The statistical model is as follows:

y(i,j,k) = m + R(i) + C(j) + F(k) + e

where y(i,j,k) is rumen degradability and intestinal digestibility, m is the population mean, R(i) is the ratio effect, C(j) is the concentrate effect, F(k) is the forage effect, and e is the random residual.

## 3. Results

### 3.1. Rumen Degradability and Intestinal Digestibility of Mutton Sheep Diets with Different Concentrate-to-Forage Ratios

**Figure 1:**
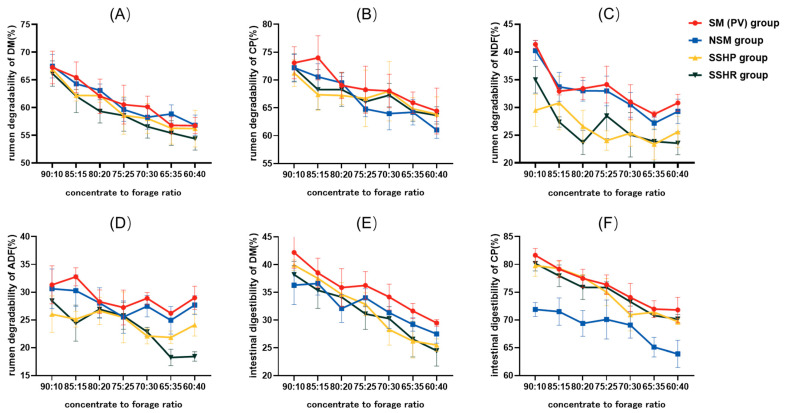
Rumen degradability and intestinal digestibility of mutton sheep diets with different concentrate-to-forage ratios. Rumen degradability of DM (**A**), CP (**B**), NDF (**C**), ADF (**D**); intestinal digestibility of DM (**E**), CP (**F**). SM (PV) group: soybean meal group (peanut vine group), raw materials: corn grain, soybean meal, DDGS, wheat bran, peanut vine, sodium bicarbonate, premix. NSM group: no soybean meal group, raw materials: corn grain, cottonseed meal, rapeseed meal, DDGS, wheat bran, peanut vine, sodium bicarbonate, premix. SSHP group: sunflower seed hull and peanut vine group, raw materials: corn grain, soybean meal, DDGS, wheat bran, sunflower seed hulls, peanut vine, sodium bicarbonate, premix. SSHR group: sunflower seed hulls and rice hulls, raw materials: corn grain, soybean meal, DDGS, wheat bran, sunflower seed hulls, rice hulls, sodium bicarbonate, premix.

The rumen degradability of DM, CP, and NDF of the four groups of experimental diets significantly decreased with the decrease in the concentrate-to-forage ratio (Figure 1A–C). The rumen degradability of ADF decreased with the decrease in the concentrate-to-forage ratio, but it was not very obvious (Figure 1D). The rumen degradability values of DM and ADF were higher when the concentrate-to-forage ratios were 75:25~90:10. The rumen degradability values of CP and NDF were higher when the concentrate-to-forage ratios were 70:30~90:10. The rumen degradability values of DM, CP, NDF, and ADF were statistically non-significant when the concentrate-to-forage ratios were 70:30~80:20.

The intestinal digestibility of the DM and CP of the four experimental diets decreased with the decrease in the concentrate-to-forage ratio (Figure 1E,F). The intestinal digestibility values of DM and CP were higher when the concentrate-to-forage ratios were 75:25~90:10. There were no differences in intestinal digestibility between DM and CP in the ratios of 75:25 and 80:20 (Figure 1E,F).

### 3.2. Rumen Degradability and Intestinal Digestibility of Mutton Sheep Diets with Different NFC/NDF Ratios

**Figure 2:**
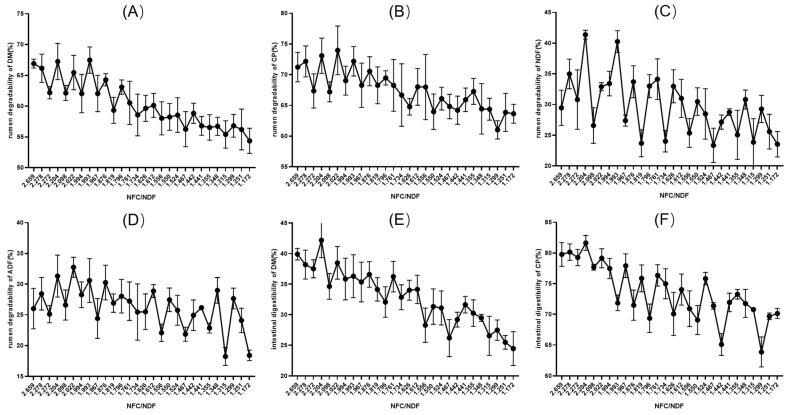
Rumen degradability and intestinal digestibility of mutton sheep diets with different NFC/NDF ratios. Rumen degradability of DM (**A**), CP (**B**), NDF (**C**), ADF (**D**); intestinal digestibility of DM (**E**), CP (**F**).

The rumen degradability of DM in the diet showed a downward trend with the decrease in NFC/NDF, and the rumen degradability values of DM in the diet at 1.761~2.659 were higher than those at 1.172~1.734, while the rumen degradability of DM in the diet at 1.524~1.994 had little change (Figure 2A). The rumen degradability of CP in the diet showed a downward trend with the decrease in NFC/NDF, and the CP rumen degradability with an NFC/NDF at 1.761~2.659 was higher than that of DM at 1.172~1.734, while the CP rumen degradability with an NFC/NDF at 1.524~1.994 had little change (Figure 2B). The rumen degradability of NDF and ADF in diets showed a downward trend with the decrease in NFC/NDF on the whole, but the NDF and ADF rumen degradability at adjacent NFC/NDF levels was significantly affected by different NFC/NDF levels in diets (Figure 2C,D).

With the decrease in dietary NFC/NDF, the intestinal digestibility of dietary DM showed a downward trend, and the intestinal digestibility of DM with the NFC/NDF at 1.612~2.659 was higher than that at 1.172~1.556. The intestinal digestibility of DM in relation to the NFC/NDF had no significant changes when it was between 1.524 and 1.994 (Figure 2E). With the decrease in dietary NFC/NDF, the intestinal digestibility of dietary CP generally showed a downward trend. The intestinal digestibility of CP with the NFC/NDF at 2.659~1.994 was higher than that with the NFC/NDF at 1.172~1.993. The intestinal digestibility of CP with NFC/NDF levels from 1.524 to 1.994 had no significant changes (Figure 2F).

### 3.3. Effects of Replacing Soybean Meal with Cottonseed Meal and Rapeseed Meal on Rumen Degradability and Intestinal Digestibility of Mutton Sheep

**Figure 3:**
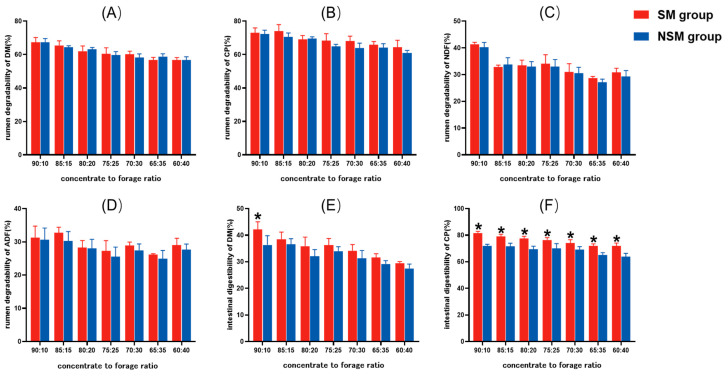
Effects of replacing soybean meal with cottonseed meal and rapeseed meal on rumen degradability and intestinal digestibility of mutton sheep. Rumen degradability of DM (**A**), CP (**B**), NDF (**C**), ADF (**D**); intestinal digestibility of DM (**E**), CP (**F**). SM group: soybean meal group, raw materials: corn grain, soybean meal, DDGS, wheat bran, peanut vine, sodium bicarbonate, premix. NSM group: no soybean meal group, raw materials: corn grain, cottonseed meal, rapeseed meal, DDGS, wheat bran, peanut vine, sodium bicarbonate, premix. * *p* < 0.05.

Rumen degradability values of DM, CP, NDF, and ADF in the SM group and NSM group were not significantly different in relation to the concentrate-to-forage ratio (*p* > 0.05) (Figure 3A–D). When the concentrate-to-forage ratio was 90:10, the intestinal digestibility of DM in the SM group was significantly higher than that in the NSM group (*p* < 0.05), and there was no significant difference between the intestinal digestibility of DM in the SM group and NSM group under other concentrate-to-forage ratios (*p* > 0.05) (Figure 3E). The CP intestinal digestibility of the SM group was significantly higher than that in the NSM group at seven concentrate-to-forage ratios (*p* < 0.05) (Figure 3F).

### 3.4. Effects of Replacing Peanut Vine with Sunflower Seed Hulls and Rice Hulls on Rumen Degradability and Intestinal Digestibility of Mutton Sheep

**Figure 4:**
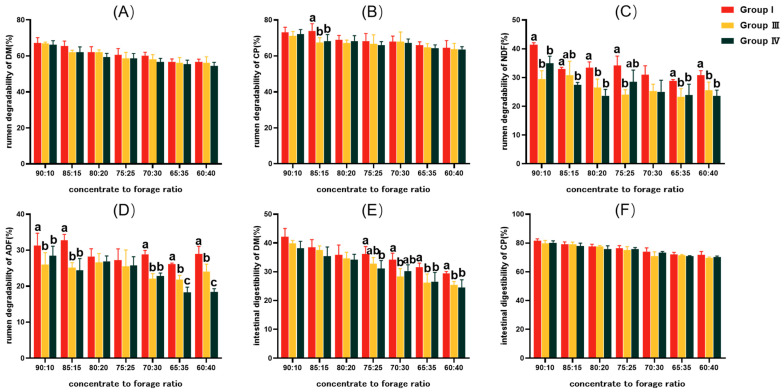
Effects of replacing peanut vine with sunflower seed hulls and rice hulls on rumen degradability and intestinal digestibility of mutton sheep. Rumen degradability of DM (**A**), CP (**B**), NDF (**C**), ADF (**D**); intestinal digestibility of DM (**E**), CP (**F**). PV group: peanut vine group, raw materials: corn grain, soybean meal, DDGS, wheat bran, peanut vine, sodium bicarbonate, premix. SSHP group: sunflower seed hull and peanut vine group, raw materials: corn grain, soybean meal, DDGS, wheat bran, sunflower seed hulls, peanut vine, sodium bicarbonate, premix. SSHR group: sunflower seed hulls and rice hulls, raw materials: corn grain, soybean meal, DDGS, wheat bran, sunflower seed hulls, rice hulls, sodium bicarbonate, premix. Different letters (a–c) indicate significant differences at *p* < 0.05.

The rumen degradability values of DM, CP, NDF, and ADF in the PV group were higher than those in the SSHP group and SSHR group under seven concentrate-to-forage ratios (Figure 4A–D). When the concentrate-to-forage ratio was 85:15, the CP rumen degradability of the PV group was significantly higher than that of the SSHP group and SSHR group (*p* < 0.05) (Figure 4B). When the concentrate-to-forage ratios were 90:10, 80:20, 65:35, and 60:40, the NDF rumen degradability of the PV group was significantly higher than that of the SSHP group and SSHR group (*p* < 0.05). When the concentrate-to-forage ratio was 85:15, the NDF rumen degradability of the PV group was significantly higher than that of the SSHR group (*p* < 0.05). When the concentrate-to-forage ratio was 75:25, the NDF rumen degradability of the PV group was significantly higher than that of the SSHR group (*p* < 0.05). The NDF rumen degradability of the PV group was significantly higher than that of the SSHP group (*p* < 0.05), but there was no significant difference between the PV group and SSHR group (*p* > 0.05) (Figure 4C). The ADF rumen degradability of the PV group was significantly higher than that of the SSHP group and SSHR group when the concentrate-to-forage ratios were 90:10 and 70:30~60:40 (*p* < 0.05), and the ADF rumen degradability of the SSHP group was significantly higher than that of the SSHR group when the concentrate-to-forage ratios were 65:35 and 60:40 (*p* < 0.05) (Figure 4D).

The intestinal digestibility of DM and CP in the PV group was higher than that in the SSHP group and SSHR group (Figure 4E,F). When the concentrate-to-forage ratio was 75:25, the rumen degradability of DM in the PV group was significantly higher than that in the SSHR group (*p* < 0.05), but there was no significant difference between the PV group and SSHP group (*p* > 0.05). When the concentrate-to-forage ratio was 70:30, the intestinal digestibility of DM in the PV group was significantly higher than that in the SSHP group (*p* < 0.05), but there was no significant difference between the PV group and SSHR group (*p* > 0.05). When the concentrate-to-forage ratios were 65:35 and 60:40, the intestinal digestibility of DM in the PV group was significantly higher than that in the SSHP group and SSHR group (*p* < 0.05). There were no significant differences in the intestinal digestibility of DM in the PV group, SSHP group, and SSHR group at other concentrate-to-forage ratios (*p* > 0.05) (Figure 4E). There were no significant differences in CP intestinal digestibility in the PV group, SSHP group, and SSHR group under seven concentrate-to-forage ratios (*p* > 0.05) (Figure 4F).

## 4. Discussion

### 4.1. Rumen Degradability and Intestinal Digestibility of Mutton Sheep Diets with Different Concentrate-to-Forage Ratios

The concentrate-to-forage ratio of the diet is the main factor affecting the production performance of ruminants. A reasonable concentrate-to-forage ratio of the diet can effectively improve the feed intake, daily gain, and digestion of ruminants, and reduce breeding costs. Within a certain range, the higher the rumen degradability and intestinal digestibility, the easier it is for the mutton sheep to use the nutrients [25]. Ewes were fed diets with ratios of 85:15, 70:30, 55:45, 40:60, 25:75, and 10:90, and it was found that the apparent digestibility of DM and OM increased with the increase in the concentrate-to-forage ratio [26]. In actual production, this concentration level is so high that it will cause acidosis, urinary calculus, and other diseases. Under normal circumstances, the pH of rumen fluid ranges from 5.5 to 7.5. When the rumen pH drops below 5.6 and persists for more than 3 h, subacute rumen acidosis (SARA) can be judged in animals [27]. Long-term rumen acidosis can lead to decreased rumen function, and can cause rumen epithelium damage and laminitis, leading to unnecessary economic losses [28]. Rumen acidosis may occur in ruminants after long-term consumption of high concentrate-to-forage-ratio diets and excessive starch intake. Reducing the proportion of roughage in ruminant diets could lead to an increase in the incidence of subacute ruminal acidosis (SARA) [4]. When Tanhan mixed sheep were fed at the fattening stage with diets with ratios of 70:30, 80:20, and 90:10 [29], after 90 days of feeding, the average daily gain of sheep in the 90:10 group was lower than that in the 80:20 group, and rumen accumulation, dyspepsia, and urinary calculi occurred [29]. The results indicate that the health of sheep is not damaged when the concentrate-to-forage ratio in the diet is below 90:10. Therefore, according to the test results of this paper, and in consideration of actual production, it is suggested to use a diet with concentrate-to-forage ratios of 70:30~80:20 during the fattening stage of mutton sheep.

### 4.2. Rumen Degradability and Intestinal Digestibility of Mutton Sheep Diets with Different NFC/NDF Ratios

With the deepening of the research, it was found that adjusting the concentrate-to-forage ratio of the diet will change the content and proportion of NFC [30], and the dietary NFC/NDF can more accurately represent the proportion of digestible carbohydrates and fiber in the diet. Picking the appropriate dietary level of the NFC/NDF in food helps ruminants grow better and stay healthy [8,9]. The nutritional regulation of ruminants by different dietary NFC/NDF levels is now a hot research topic [31]. In this experiment, the rumen degradability values of DM and CP were higher when the NFC/NDF was in the range of 1.761~2.659. The digestibility of DM was higher when the NFC/NDF was in the range of 1.612~2.659. The digestibility of CP was higher at levels of 1.994~2.659. Many studies have proved that the dietary NFC/NDF can improve the performance of animals [32,33], but an exorbitant NFC/NDF will reduce the growth rate, which is not conducive to rumen health. Some researchers used diets with NFC/NDF ratios of 1.43, 1.63, 1.88, 2.06, 2.42, 2.69, and 2.86 for fattening Tibetan sheep. The experiment showed that an NFC/NDF of 2.69 showed the fastest growth rate and the highest TVFA content, but when the NFC/NDF reached 2.86, the growth rate decreased [34]. The highest NFC/NDF ratio in this study was lower than what would cause rumen acidosis. In addition, in this experiment, the rumen degradability and intestinal digestibility of DM and CP had little change when the NFC/NDF ranged from 1.524 to 1.994, and the rumen degradability and intestinal digestibility of DM and CP also had little change when the NFC/NDF ranged from 2.022 to 2.659. When the NFC/NDF ranged from 2.022 to 2.659, the corresponding concentrate-to-forage ratio was basically more than 85:15. Combined with the experimental results of the concentrate-to-forage ratio, it is suggested to use a diet with an NFC/NDF of 1.5~2.0 during the fattening stage of mutton sheep.

### 4.3. Effects of Replacing Soybean Meal with Cottonseed Meal and Rapeseed Meal on Rumen Degradability and Intestinal Digestibility of Mutton Sheep

In recent years, the price of soybean meal has been high, which has made the price of the diet rise. Without affecting the growth performance of sheep, it is of great significance to find a low-cost protein feed to replace soybean meal to reduce the price of the diet and improve the economic benefits of farmers. The prices of cottonseed meal and rapeseed meal are lower than that of soybean meal, so replacing soybean meal with cottonseed meal can greatly reduce the cost of the diet and improve the economic benefit of animal husbandry [13]. In this study, under seven different concentrate-to-forage ratios, the rumen degradability of DM, CP, NDF, and ADF did not differ significantly between the SM group and the NSM group. Many researchers have found through in situ methods that the rumen degradability of soybean meal is significantly higher than those of cottonseed meal and rapeseed meal [35,36]. This study is different in its research results. The difference may be due to the fact that the rumen degradability of a single feed material was measured in the former test, whereas in this test, the rumen degradability of a mixed diet was measured. The maximum inclusion level of soybean meal, cottonseed meal, and rapeseed meal was 14%. Aside from the different protein feeds, the other feed materials and their proportions in the diet were the same. Thus, the significant differences in the rumen degradability of soybean meal, cottonseed meal, and rapeseed meal were neutralized. The intestinal digestibility of the NSM group was significantly lower than that of the SM group, which may be due to the anti-nutritional factors, such as gossypol, tannin, and phytic acid, contained in cottonseed meal and rapeseed meal [37]. However, the digestion and absorption of nutrients in the diet of ruminants is mainly carried out in the rumen [38]. Moreover, the feasibility of replacing soybean meal with cottonseed meal and rapeseed meal has been verified in animal experiments that have been conducted. Studies on Hu sheep have shown that replacing soybean meals with fermented cottonseed meals and rapeseed meals can improve growth performance and energy utilization efficiency, with no observed negative effects on rumen microbiota [13]. Similarly, feeding rams with rapeseed meal instead of soybean meal resulted in no significant differences in growth performance and DM digestibility [39]. The addition of cottonseed meal did not affect the production performance of sheep, and it reduced the cost of diets [40]. Based on the findings of this study, cottonseed meal and rapeseed meal can serve as viable alternatives to soybean meal as the primary protein source for mutton sheep, particularly when the price of soybean meal is high and farming efficiency is low; however, this substitution may reduce the intestinal digestibility of dietary CP.

### 4.4. Effects of Replacing Peanut Vine with Sunflower Seed Hulls and Rice Hulls on Rumen Degradability and Intestinal Digestibility of Mutton Sheep

Roughage is an important part of the diet; choosing the appropriate roughage can not only improve the production performance of animals, but also reduce the use of concentrate feed to a certain extent [41]. In this experiment, there was no significant difference in the rumen degradability of DM in the PV group, SSHP group, and SSHR group, but on the whole, the results were as follows: PV group > SSHP group > SSHR group. Further, the rumen degradability of CP was also the highest in the PV group. The rumen degradability of NDF and ADF and the intestinal digestibility of DM in the PV group were significantly higher than those in the SSHP group and SSHR group under most of the concentrate-to-forage ratios. This indicates that the digestibility of the nutrients of mutton sheep when the roughage is peanut vine is higher than that when the roughage is 50% sunflower seed hulls instead of peanut vine and when sunflower seed hulls and rice hulls completely replace peanut vine. At present, there are few studies on sunflower seed hulls and rice hulls, but there are a lot of studies on the peanut vine. It was found that the supplementation of peanut vine in millet stover diets for ruminants improved production performance and effectively reduced feeding costs [42], and replacing wheat straw with peanut vine could improve the growth performance, rumen fermentation, meat quality, and microbiota of cattle [43]. These studies show that peanut vine is a kind of high-quality roughage for mutton sheep raising, which is conducive to the growth and development of ruminants. The results this study showed are similar to the results of previous studies. In addition, the rumen degradability and intestinal digestibility of DM and CP and the rumen degradability of NDF in the SSHP group and SSHR group were not significantly different under the seven concentrate-to-forage ratios. The rumen degradability of ADF in the SSHP group was significantly higher than that in the SSHR group when the concentrate-to-forage ratios were 65:35 and 60:40, but there was no significant difference in other groups. These results indicate that the roughage of a sunflower seed hull and peanut vine diet has a higher feeding value than that of a sunflower seed hull and rice hull diet. In conclusion, the nutritional value of sunflower seed hulls and rice hulls fed to mutton sheep is lower than that of peanut vine. When the price is reasonable, peanut vine should be selected as often as possible when formulating diets for mutton sheep.

## 5. Conclusions

Concentrate-to-forage ratios of 70:30~80:20 and NFC/NDF ratios of 1.5~2.0 are recommended for fattening mutton sheep. In addition, low-cost protein feed (cottonseed meal and rapeseed meal) can be feasible alternatives to soybean meal. The nutritional values of sunflower seed hulls and rice hulls for mutton sheep were lower than that of peanut vine. The dietary concentrate level and feed material selection obtained in this experiment are of great significance to the high-quality development of the mutton sheep industry.

## Figures and Tables

**Table 1:** Dietary composition and nutrient levels (air-dry basis).

Ingredients	Content
Concentrate feed ^1^ (%)	21.69
Corn stalk silage (%)	48.19
Weeds (%)	30.12
Total (%)	100
Nutrient composition	
DM (%)	88.76
CP (%)	9.13
EE (%)	4.63
NDF (%)	54.28
ADF (%)	17.82
Ca (%)	0.35
P (%)	0.23
Ca/P	1.52

^1^ The main components of concentrate feed: corn grain, soybean meal, cottonseed meal, DDGS (distillers’ dried grains with solubles), corn gluten meal, CaHPO_4_ (Calcium Perphosphate), CaCO_3_ (Calcium Carbonate), MgO (Magnesium Oxide), NaCl (Sodium Chloride). Trace elements, minerals, and vitamins were supplemented in the form of lick blocks. The composition of the lick blocks was Cu (Copper), Fe (Iron), Zn (Zinc), Mn (Manganese), Se (Selenium), Co (Cobalt), I (Iodine), Vitamin E, Vitamin A. All abbreviations: DM (Dry Matter), CP (Crude Protein), EE (Ether Extract), NDF (Neutral Detergent Fiber), ADF (Acid Detergent Fiber), Ca (Calcium), P (Phosphorus), Ca/P (Calcium/Phosphorus).

**Table 2:** SM (PV) group experimental diet raw material composition and nutritional level (air-dry basis).

SM (PV) Group	Concentrate-to-Forage Ratio						
Raw Material	90:10	85:15	80:20	75:25	70:30	65:35	60:40
Corn grain (%)	40.00	38.00	36.00	34.00	32.00	30.00	28.00
Soybean meal (%)	14.00	13.00	12.00	11.00	10.00	9.00	8.00
DDGS (%)	14.00	13.00	12.00	11.00	10.00	9.00	8.00
Wheat bran (%)	16.00	15.00	14.00	13.00	12.00	11.00	10.00
Peanut vine (%)	10.00	15.00	20.00	25.00	30.00	35.00	40.00
Sodium bicarbonate (%)	1.00	1.00	1.00	1.00	1.00	1.00	1.00
Premix (%) ^1^	5.00	5.00	5.00	5.00	5.00	5.00	5.00
Total (%)	100.00	100.00	100.00	100.00	100.00	100.00	100.00
Nutrient composition							
DE (MJ/kg) ^2^	13.23	12.95	12.68	12.41	12.13	11.86	11.58
ME (MJ/kg) ^2^	10.89	10.66	10.44	10.21	9.99	9.76	9.54
DM (%)	93.73	92.25	92.54	93.10	93.34	94.29	94.48
NDF (%)	21.69	23.62	24.30	26.56	28.25	30.70	32.11
ADF (%)	10.46	12.18	13.53	15.42	17.47	19.82	21.47
CP (%)	19.16	17.87	16.46	16.08	15.33	14.74	13.57
EE (%)	3.74	3.32	3.40	3.03	3.10	2.24	2.71
Ash (%)	7.60	7.42	7.39	7.58	7.77	8.08	8.33
Ca (%)	1.19	1.21	1.25	1.26	1.29	1.36	1.38
P (%)	0.69	0.63	0.61	0.59	0.59	0.57	0.57
NFC (%) ^3^	47.82	47.78	48.46	46.75	45.54	44.25	43.28
NFC/NDF	2.204	2.022	1.994	1.761	1.612	1.441	1.348
Ca/P	1.73	1.94	2.06	2.12	2.20	2.40	2.41

SM (PV) group: soybean meal group (peanut vine group), raw materials: corn grain, soybean meal, DDGS, wheat bran, peanut vine, sodium bicarbonate, premix. All abbreviations: DE (Digestible Energy), ME (Metabolizable Energy), DM (Dry Matter), NDF (Neutral Detergent Fiber), ADF (Acid Detergent Fiber), CP (Crude Protein), EE (Ether Extract), Ash (Crude ash), Ca (Calcium), P (Phosphorus), NFC (Nonfiber Carbohydrates), NFC/NDF (Nonfiber Carbohydrates/Neutral Detergent Fiber), Ca/P (Calcium/Phosphorus). ^1^ Each kilogram of premix contained the following: Vitamin A: 150,000–300,000 IU, Vitamin D_3_: 40,000–10,000 IU, Vitamin E: 450–1200 IU, Cu: 0.05–0.6 g, Fe: 0.5–2 g, Zn: 1–3 g, Mn: 0.6–2 g, I: 6–60 mg, Se: 2–20 mg, Co: 6–30 mg, Ca: 10–25%, P: 2–5%, NaCl: 10%, hydration ≤ 10%. ^2^ DE and ME were calculated; the calculation was based on the “Nutrient requirements of mutton sheep and goat (NY/T 816-2021)”; others are measured values. ^3^ NFC = 100 – (NDF + CP + EE + Ash).

**Table 3:** NSM group experimental diet raw material composition and nutritional level (air-dry basis).

NSM Group	Concentrate-to-Forage Ratio						
Raw Material	90:10	85:15	80:20	75:25	70:30	65:35	60:40
Corn grain (%)	40.00	38.00	36.00	34.00	32.00	30.00	28.00
Cottonseed meal (%)	5.00	5.00	4.00	4.00	4.00	3.00	2.00
DDGS (%)	14.00	13.00	12.00	11.00	10.00	9.00	8.00
Rapeseed meal (%)	9.00	8.00	8.00	7.00	6.00	6.00	6.00
Wheat bran (%)	16.00	15.00	14.00	13.00	12.00	11.00	10.00
Peanut vine (%)	10.00	15.00	20.00	25.00	30.00	35.00	40.00
Sodium bicarbonate (%)	1.00	1.00	1.00	1.00	1.00	1.00	1.00
Premix (%) ^1^	5.00	5.00	5.00	5.00	5.00	5.00	5.00
Total (%)	100.00	100.00	100.00	100.00	100.00	100.00	100.00
Nutrient composition							
DE (MJ/kg) ^2^	12.87	12.62	12.37	12.12	11.88	11.62	11.37
ME (MJ/kg) ^2^	10.59	10.39	10.18	9.98	9.78	9.57	9.37
DM (%)	92.65	92.92	94.20	94.69	93.28	94.80	95.13
NDF (%)	23.68	25.08	26.43	28.27	29.56	31.27	33.58
ADF (%)	11.69	13.42	14.91	16.64	18.34	19.74	22.47
CP (%)	17.16	16.08	14.80	14.08	13.37	12.92	11.27
EE (%)	4.70	4.39	3.83	3.91	3.59	3.85	3.78
Ash (%)	7.26	7.40	7.49	7.77	7.67	7.88	8.10
Ca (%)	1.14	1.15	1.22	1.230	1.25	1.34	1.39
P (%)	0.73	0.72	0.68	0.65	0.63	0.60	0.58
NFC (%) ^3^	47.20	47.05	47.46	45.98	45.82	45.08	43.63
NFC/NDF	1.993	1.876	1.796	1.626	1.550	1.442	1.299
Ca/P	1.55	1.60	1.79	1.89	1.99	2.22	2.41

NSM group: No soybean meal group, raw materials: corn grain, cottonseed meal, rapeseed meal, DDGS, wheat bran, peanut vine, sodium bicarbonate, premix. All abbreviations: DE (Digestible Energy), ME (Metabolizable Energy), DM (Dry Matter), NDF (Neutral Detergent Fiber), ADF (Acid Detergent Fiber), CP (Crude Protein), EE (Ether Extract), Ash (Crude ash), Ca (Calcium), P (Phosphorus), NFC (Nonfiber Carbohydrates), NFC/NDF (Nonfiber Carbohydrates/Neutral Detergent Fiber), Ca/P (Calcium/Phosphorus). ^1^ Each kilogram of premix contained the following: Vitamin A: 150,000–300,000 IU, Vitamin D_3_: 40,000–10,000 IU, Vitamin E: 450–1200 IU, Cu: 0.05–0.6 g, Fe: 0.5–2 g, Zn: 1–3 g, Mn: 0.6–2 g, I: 6–60 mg, Se: 2–20 mg, Co: 6–30 mg, Ca: 10–25%, P: 2–5%, NaCl: 10%, hydration ≤ 10%. ^2^ DE and ME were calculated; the calculation was based on the “Nutrient requirements of mutton sheep and goat (NY/T 816-2021)”; others are measured values. ^3^ NFC = 100 – (NDF + CP + EE + Ash).

**Table 4:** SSHP group experimental diet raw material composition and nutritional level (air-dry basis).

SSHP Group	Concentrate-to-Forage Ratio						
Raw Material	90:10	85:15	80:20	75:25	70:30	65:35	60:40
Corn grain (%)	40.00	38.00	36.00	34.00	32.00	30.00	28.00
Soybean meal (%)	14.00	13.00	12.00	11.00	10.00	9.00	8.00
DDGS (%)	14.00	13.00	12.00	11.00	10.00	9.00	8.00
Wheat bran (%)	16.00	15.00	14.00	13.00	12.00	11.00	10.00
Sunflower seed hulls (%)	5.00	10.00	10.00	15.00	15.00	20.00	20.00
Peanut vine (%)	5.00	5.00	10.00	10.00	15.00	15.00	20.00
Sodium bicarbonate (%)	1.00	1.00	1.00	1.00	1.00	1.00	1.00
Premix (%) ^1^	5.00	5.00	5.00	5.00	5.00	5.00	5.00
Total (%)	100.00	100.00	100.00	100.00	100.00	100.00	100.00
Nutrientcomposition							
DE (MJ/kg) ^2^	13.19	12.89	12.61	12.30	12.03	11.72	11.45
ME (MJ/kg) ^2^	10.86	10.61	10.38	10.13	9.90	9.65	9.43
DM (%)	94.03	92.85	94.62	94.96	94.60	93.20	95.61
NDF (%)	19.61	22.20	23.48	27.25	29.59	31.12	34.18
ADF (%)	9.611	11.86	13.27	16.68	18.71	20.20	22.80
CP (%)	16.29	15.92	15.25	14.69	14.06	13.57	12.68
EE (%)	4.78	4.41	4.88	3.67	3.17	2.42	2.99
Ash (%)	7.19	7.01	7.14	7.13	7.15	7.21	7.41
Ca (%)	1.09	1.12	1.14	1.18	1.19	1.20	1.20
P (%)	0.66	0.65	0.65	0.65	0.62	0.60	0.60
NFC (%) ^3^	52.14	50.45	49.25	47.25	46.03	45.67	42.75
NFC/NDF	2.659	2.272	2.098	1.734	1.556	1.467	1.251
Ca/P	1.64	1.71	1.77	1.81	1.91	1.98	1.99

SSHP group: sunflower seed hull and peanut vine group, raw materials: corn grain, soybean meal, DDGS, wheat bran, sunflower seed hulls, peanut vine, sodium bicarbonate, premix. All abbreviations: DE (Digestible Energy), ME (Metabolizable Energy), DM (Dry Matter), NDF (Neutral Detergent Fiber), ADF (Acid Detergent Fiber), CP (Crude Protein), EE (Ether Extract), Ash (Crude ash), Ca (Calcium), P (Phosphorus), NFC (Nonfiber Carbohydrates), NFC/NDF (Nonfiber Carbohydrates/Neutral Detergent Fiber), Ca/P (Calcium/Phosphorus). ^1^ Each kilogram of premix contained the following: Vitamin A: 150,000–300,000 IU, Vitamin D_3_: 40,000–10,000 IU, Vitamin E: 450–1200 IU, Cu: 0.05–0.6 g, Fe: 0.5–2 g, Zn: 1–3 g, Mn: 0.6–2 g, I: 6–60 mg, Se: 2–20 mg, Co: 6–30 mg, Ca: 10–25%, P: 2–5%, NaCl: 10%, hydration ≤ 10%. ^2^ DE and ME were calculated; the calculation was based on the “Nutrient requirements of mutton sheep and goat (NY/T 816-2021)”; others are measured values. ^3^ NFC = 100 − (NDF + CP + EE + Ash).

**Table 5:** SSHR group experimental diet raw material composition and nutritional level (air-dry basis).

SSHR Group	Concentrate-to-Forage Ratio						
Raw Material	90:10	85:15	80:20	75:25	70:30	65:35	60:40
Corn grain (%)	40.00	38.00	36.00	34.00	32.00	30.00	28.00
Soybean meal (%)	14.00	13.00	12.00	11.00	10.00	9.00	8.00
DDGS (%)	14.00	13.00	12.00	11.00	10.00	9.00	8.00
Wheat bran (%)	16.00	15.00	14.00	13.00	12.00	11.00	10.00
Sunflower seed hulls (%)	5.00	10.00	10.00	15.00	15.00	20.00	20.00
Rice hulls (%)	5.00	5.00	10.00	10.00	15.00	15.00	20.00
Sodium bicarbonate (%)	1.00	1.00	1.00	1.00	1.00	1.00	1.00
Premix (%) ^1^	5.00	5.00	5.00	5.00	5.00	5.00	5.00
Total (%)	100.00	100.00	100.00	100.00	100.00	100.00	100.00
Nutrient composition							
DE (MJ/kg) ^2^	13.07	12.76	12.36	12.05	11.66	11.35	10.95
ME (MJ/kg) ^2^	10.76	10.51	10.18	9.93	9.60	9.35	9.02
DM (%)	92.53	90.02	90.85	93.09	94.29	93.09	94.57
NDF (%)	21.54	24.45	25.51	28.86	30.75	32.75	34.18
ADF (%)	10.11	12.45	13.56	16.24	17.90	19.76	20.91
CP (%)	16.61	16.07	15.51	14.59	14.42	12.88	12.71
EE (%)	5.19	4.15	4.57	4.43	4.20	2.43	3.21
Ash (%)	7.59	7.23	8.01	8.12	8.98	8.89	9.84
Ca (%)	1.07	1.06	1.06	1.06	1.07	1.10	1.13
P (%)	0.71	0.69	0.67	0.65	0.65	0.64	0.64
NFC (%) ^3^	49.08	48.10	46.41	43.99	41.65	43.05	40.07
NFC/NDF	2.278	1.967	1.819	1.524	1.355	1.315	1.172
Ca/P	1.50	1.54	1.58	1.62	1.66	1.72	1.77

SSHR group: sunflower seed hulls and rice hulls, raw materials: corn grain, soybean meal, DDGS, wheat bran, sunflower seed hulls, rice hulls, sodium bicarbonate, premix. All abbreviations: DE (Digestible Energy), ME (Metabolizable Energy), DM (Dry Matter), NDF (Neutral Detergent Fiber), ADF (Acid Detergent Fiber), CP (Crude Protein), EE (Ether Extract), Ash (Crude ash), Ca (Calcium), P (Phosphorus), NFC (Nonfiber Carbohydrates), NFC/NDF (Nonfiber Carbohydrates/Neutral Detergent Fiber), Ca/P (Calcium/Phosphorus). ^1^ Each kilogram of premix contained the following: Vitamin A: 150,000–300,000 IU, Vitamin D_3_: 40,000–10,000 IU, Vitamin E: 450–1200 IU, Cu: 0.05–0.6 g, Fe: 0.5–2 g, Zn: 1–3 g, Mn: 0.6–2 g, I: 6–60 mg, Se: 2–20 mg, Co: 6–30 mg, Ca: 10–25%, P: 2–5%, NaCl: 10%, hydration ≤ 10%. ^2^ DE and ME were calculated; the calculation was based on the “Nutrient requirements of mutton sheep and goat (NY/T 816-2021)”; others are measured values. ^3^ NFC = 100 – (NDF + CP + EE + Ash).

## Data Availability

The data used to support the findings of this study will not be publicly available as we will be conducting further research.
